# Crocetin Exerts Its Anti-inflammatory Property in LPS-Induced RAW264.7 Cells Potentially via Modulation on the Crosstalk between MEK1/JNK/NF-*κ*B/iNOS Pathway and Nrf2/HO-1 Pathway

**DOI:** 10.1155/2021/6631929

**Published:** 2021-09-10

**Authors:** Yi-Ling Wen, Ziyu He, De-Xing Hou, Si Qin

**Affiliations:** ^1^Key Laboratory for Food Science and Biotechnology of Hunan Province, College of Food Science and Technology, Hunan Agricultural University, Changsha 410128, China; ^2^The United Graduate School of Agricultural Sciences, Faculty of Agriculture, Kagoshima University, Korimoto 1-21-24, Kagoshima 890-0065, Japan

## Abstract

Crocetin is a main bioactive component with a carotenoid skeleton in *Gardenia jasminoides*, a typical traditional Chinese medicine with a long history in Southeast Asia. Crocetin is being commonly consumed as spices, dyes, and food colorants. Recent pharmacological studies had implied that crocetin may possess potent anti-inflammatory properties; however, the underlying molecular mechanism is not fully elucidated. In the present study, the regulatory effect of crocetin on redox balance was systematically investigated in lipopolysaccharide- (LPS-) stimulated RAW264.7 cells. The results showed that crocetin dose-dependently inhibited LPS-induced nitric oxide production and inducible nitric oxide synthase (iNOS) expression in RAW264.7 cells. Molecular data revealed that crocetin exerted its anti-inflammatory property by inhibiting the MEK1/JNK/NF-*κ*B/iNOS pathway and activating the Nrf2/HO-1 pathway. The shRNA-knockdown (KD) of MEK1 and ERK1 confirmed that the activation of MEK1 and inhibition of JNK mediated the anti-inflammatory effect of crocetin. Moreover, the pull-down assay and computational molecule docking showed that crocetin could directly bind to MEK1 and JNK1/2. It is noticed that both KD and knockout (KO) of *HO-1* gene blocked this action. More detailed data have shown that *HO-1*-KO blocked the inhibition of p-I*κ*B-*α* by crocetin. These data indicated that crocetin exerted its anti-inflammatory property via modulating the crosstalk between the MEK1/JNK/NF-*κ*B/iNOS pathway and the Nrf2/HO-1 pathway, highlighting HO-1 as a major player. Therefore, the present study reveals that crocetin can act as a potential candidate for redox-balancing modulation in charge of its anti-inflammatory and chemopreventive effect, which strengthens its potency in the subsequent clinic application in the near future.

## 1. Introduction

*Gardenia jasminoides* is widely planted in China, Japan, Korea, India, and North America [1]. As a traditional and crucial herbal medicine, *Gardenia jasminoides* is recorded in Chinese Pharmacopoeia, with multiple functions on clearing heat and detoxifying, reducing fever and headache, causing diuresis, cooling blood, and eliminating swelling [2, 3]. Plenty of pharmacological studies had found that *Gardenia jasminoides* can possess various pharmacological activities in hepatochemoprevention [4, 5], neuroprotection [6], anti-inflammatory regulation [7, 8], and antithrombotic and cardiovascular intervention [1, 9, 10], In addition, it is also widely applied as an excellent natural colorant. Crocetin, as the major natural colorant in *Gardenia jasminoides*, has been used in many fields, such as cotton, textiles, beverages, and food. The beneficial characteristics and pharmacological activities of crocetin have also been reported previously, including the chemopreventive effect on tumors [11–13], neurodegeneration [14–16], cardiovascular disease [17, 18], hepatic damage [19, 20], diabetes [21–23], and colitis diseases [24]. Molecular mechanism studies suggested that crocetin's biological activity may be attributed to its anti-inflammatory ability [14, 25]. However, the underlying molecular mechanism of its anti-inflammatory ability has not been fully elucidated.

Nitric oxide (NO), a highly reactive gas signal molecule, is involved in various physiological and pathological processes in many organ systems including nervous, immune, and cardiovascular [26, 27]. The production of NO is precisely regulated by nitric oxide synthase (NOS). There are three isozymes of NOS: the two constitutively expressed forms, endothelial NOS (eNOS) and neuronal NOS (nNOS), and the inducible form (iNOS) [28]. Significantly, high-level iNOS expression is associated with various inflammations and chronic diseases, such as stroke, demyelinating diseases, cancer, and neurodegenerative diseases [28, 29]. Thus, identification of iNOS inhibitors is considered to be a promising approach to prevent acute and chronic inflammatory states.

The nuclear factor erythroid 2 related factor 2 (Nrf2)/heme oxygenase 1 (HO-1) signaling pathway plays a critical role in protecting cells against excess oxidative stress stimuli, and it can maintain intracellular redox homeostasis via regulation on a variety of pathways such as autophagy, ferroptosis, pyroptosis, and apoptosis [30]. Recent molecular evidence shows that the Nrf2/HO-1 signaling pathway plays important role on the anti-inflammatory property of bioactive compounds [31], and overexpression of HO-1 in macrophages can significantly inhibit the production of proinflammatory cytokines by LPS stimulation and increase the anti-inflammatory response [32]. Further *in vivo* data shows that IL-10-mediated protection against LPS-induced septic shock in mice was significantly attenuated by cotreatment with the HO-1 inhibitor, zinc protoporphyrin [33], and both mice and humans lacking HO-1 expression have a phenotype that develops a chronic inflammatory state [34, 35]. These data imply that there exists a key link between antioxidation and anti-inflammation, and activation of Nrf2/HO-1 may be the potent and promising new molecular target for the treatment of inflammation-related disorders. Moreover, the crosstalk between the Nrf2/HO-1 pathway and the inflammatory pathway has been investigated in several studies, and HO-1 is a candidate to connect these pathways [36, 37]. However, this crosstalk is not well studied and lacks in-depth molecular evidence.

Therefore, in the present study, the molecular mechanism underlying the anti-inflammatory and antioxidant effect of crocetin is fully investigated by the application of shRNA-KD and CRISPR-Cas9-KO technologies in LPS-induced inflammatory RAW264.7 cells, especially the role of crocetin on modulation of redox balance, the crosstalk between the Nrf2/HO-1 pathway and the inflammatory pathway, and its targeted extracellular receptors.

## 2. Materials and Methods

### 2.1. Materials and Cell Culture

Crocetin is donated by Tairui Biotechnology Co., Ltd (Nanyang, Henan, China). Crocetin was purified by HPLC with 98% purity, and it can be dissolved in DMSO (0.1% final concentration in culture medium). LPS (*Escherichia coli* Serotype 055:B5) was purchased from Sigma (St. Louis, MO, USA). AZD6244, SB202190, SP600125, and U0126 were from MedChemExpress (Monmouth Junction, NJ, USA). Antibodies against iNOS, phospho-ERK1/2, phospho-p38 kinase, phospho-JNK, ERK1/2, p38 kinase, JNK, and I*κ*B-*α* were purchased from Cell Signaling Technology (Beverly, MA, USA). GAPDH and p65 were from Santa Cruz Biotechnology, Inc. (Santa Cruz, CA, USA). Antibodies against HO-1 and Nrf2 were from Abcam (Cambridge, MA, USA); 2′,7′-dichlorofluorescein diacetate (DCFH-DA) was from Beyotime Institute of Biotechnology (Beyotime, Guangzhou, China). Fetal bovine serum (FBS) was from Biological Industries (Kibbutz Beit Haemek, Israel). Murine macrophage-like RAW264.7 cells were purchased from ATCC (Rockville, MD, USA) and cultured at 37°C in a 5% CO_2_ atmosphere in Dulbecco's modified Eagle's medium (DMEM) containing 10% FBS. Because FBS contains numerous compounds, such as LPS and growth factors, which influence the biological characteristics of macrophages [38–41], we performed all of the experiments under serum-free conditions.

### 2.2. Cell Viability Assay

The cell viability was measured by an MTT assay. Briefly, RAW264.7 cells were seeded in a 96-well plate and treated with various concentrations of crocetin for 24 h. Subsequently, the supernatant was discarded, and 50 *μ*l of MTT solution was added to each well. Cells were incubated at 37°C for an additional 4 h in the dark. The acidic isopropanol was added to dissolve the formazan crystals, and the optical density (OD) was measured at 570 nm with a microplate reader (Thermo Scientific, MA, USA). Viability was determined by comparing the OD of sample-treated cells with those of the untreated cells.

### 2.3. Pull-Down Assay

Crocetin-Sepharose 4B beads were prepared as described previously [42]. Briefly, crocetin (5 mg) was coupled to CNBr-activated Sepharose 4B beads (75 mg) in a coupling buffer overnight at 4°C according to the manufacturer's instructions. The mixture was washed in 5 volumes of coupling buffer and then centrifuged at 110 g for 3 min at 4°C. The precipitate was resuspended in 5 volumes of 0.1 M Tris-HCl buffer (pH 8.0) with 2 h rotation at room temperature. After washing three times with 0.1 M acetate buffer (pH 4.0) containing 0.5 M NaCl, the mixture was further washed with 0.1 M Tris-HCl (pH 8.0) buffer containing 0.5 M NaCl. The RAW264.7 cell lysate (500 g/mL) was incubated at 4°C overnight with Sepharose 4B beads or Sepharose 4B crocetin-coupled beads (100 *μ*l, 50% slurry) in reaction buffer (NP-40). The beads were then washed five times with a washing buffer. The proteins were detected by Western blotting with each specific antibody.

### 2.4. Molecular Modeling

The modeling of crocetin to TLR4-MD2, MEK1, JNK1/2, and Keap1-Nrf2 proteins (PDB codes: 5IJB, 1S9J, 3V3V, and 3WN7) was performed using Molecular Operating Environment™ software (MOE, Version 2012.10, Chemical Computing Group Inc.) [43]. Hydrogen atoms were first added, and force field atomic charges were assigned. Docking of crocetin to protein kinases was done by using MOE-AS EDock 2013 software [42].

### 2.5. Construction and Transfection of Short Hairpin RNA (shRNA)

Three short hairpin RNA (shRNA) interference sequences targeting mouse HO-1, MEK1, ERK1, Nrf2, and a negative control sequence (NC) were designed, synthesized, and cloned into the pLKO.1 vector. After DNA sequencing, a three-plasmid-based lentiviral packaging system (vector plasmid-psPAX2-pMD2.G) was used to transfect HEK293T cells to package lentiviruses. Plasmids were transiently transfected into HEK293T cells using the Lipofectamine 3000 DNA Transfection Reagent (Thermo Scientific, MA, USA). The supernatants containing viruses were harvested and added with PGE8000 at a final concentration of 5% for incubation overnight and then were centrifuged at 7000 g for 20 min at 4°C by ultracentrifugation (Thermo Scientific, MA, USA). RAW264.7 cells were incubated with lentivirus for at least 36 h for successful infection. Puromycin was added for screening living cells, and the interference efficiency was detected by Western blotting.

### 2.6. Construction of *HO-1* Knockout Gene by CRISPR System

Single-guide RNA (sgRNA) targeting mouse *HO-1* gene (NM_010442.2) was designed using the CRISPR design website (http://crispr.mit.edu, in the public domain). The control sgRNA sequence (5′-TGCGAATACGCCCACGCGATGGG-3′) was designed to target the lacZ gene from *Escherichia coli* [44]. The lentiCRISPR-v2 vector was purchased from Addgene (Cat. 52961; Cambridge, MA, USA). To express sgRNA in the RAW264.7 cells, the oligos of top oligos 5′-CACCG-20 nt and bottom oligos 5′-AAAC-20 nt-C-3′ were annealed and cloned into the lentiCRISPR-v2 vector by BsmB1 (New England Biolabs, Boston, MA, USA). The flowing steps (virus packaging, virus infection, and puromycin screening) were done as described in shRNA-KD. Subsequently, the monoclonal cells were picked out and cultured into clumps, and the knockout efficiency was detected by Western blotting.

### 2.7. Measurement of Nitric Oxide (NO) and Intracellular ROS Assay

Nitric oxide in the culture medium is stably present as nitrite (NO_2_-) which can be assayed by using the Griess reagent (Madison, WI, USA). According to the manufacturer's manual, RAW264.7 cells (1.0 × 10^6^ cells) were seeded into each well of 12-well plates. After preculture for 24 h, cells were starved by being cultured in serum-free medium for another 2.5 h to eliminate the influence of FBS. The cells were then treated with or without crocetin for 1.5 h before exposure to 40 ng/ml LPS for 12 h. The level of nitric oxide released into the culture medium was determined by measuring absorbance at 530 nm in a microplate reader.

The intracellular reactive oxygen species (ROS) generation of cells was investigated using DCFH-DA as a well-established compound to detect and quantify intracellular-produced hydrogen peroxide [45]. In brief, RAW264.7 cells were plated in a 96-well plate and pretreated with crocetin for 2 h before exposure to LPS (40 ng/ml) for 12 h. DCFH-DA was added to the medium with a final concentration of 20 *μ*M for an additional 30 min. The fluorescence intensity was then measured at an excitation (485 nm) and emission (530 nm) wavelength using a fluorescent Multilabel Counter (Perkin-Elmer) and was expressed as the percentage of DMSO vehicle control in the absence of LPS. Data are the mean ± SD of three separate experiments.

### 2.8. Nuclear Protein Extraction and Western Blot Analysis

Nuclear extracts were prepared according to the manufacturer's manual (Sigma, St. Louis, MO, USA). Harvested cells were lysed in buffer A on ice for 10 min and then centrifuged at 17000 g for 10 min at 4°C. The nuclear pellets were resuspended in buffer B on ice for 40 min and then centrifuged at 17000 g for 10 min at 4°C. The supernatants containing nuclear extracts were stored at –80°C until use.

Protein concentration was determined by using a dye-binding protein assay kit (Beyotime, Guangzhou, China). Protein extracts were separated by 10% SDS-PAGE and transferred to PVDF membranes (Amersham Pharmacia Biotech, Little Chalfont, UK). The membranes were blocked at room temperature for 1 h with 5% nonfat dry milk and then incubated with each primary antibody at 4°C overnight and further incubated for 1 h with HRP-conjugated secondary antibody. Bound antibodies were detected by the ECL system with a Lumi Vision PRO machine.

### 2.9. Statistical Analyses

ImageJ and Prism 5.0 were utilized to perform quantitative analysis. All of the results from three independent experiments and data were expressed as the mean ± standard error of the mean (SEM) for the number of experiments. A statistical probability of *p* < 0.05 was considered statistically significant.

## 3. Results

### 3.1. Crocetin Suppresses LPS-Induced NO Release and iNOS Expression

**Figure 1:**
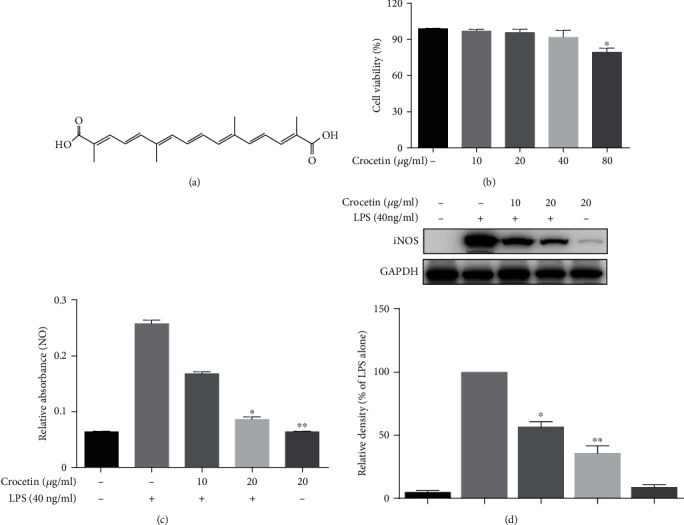
The effects of crocetin on nitric oxide production and iNOS expression. (a) The structure of crocetin. (b) The cytotoxicity assay of crocetin in RAW264.7 cells. Cytotoxicity assay was performed as described in Materials and Methods. (c) Crocetin inhibits LPS-induced NO release. RAW264.7 cells were treated with crocetin (10–20 *μ*g/ml) for 1 h before starvation in serum-free medium for another 2.5 h and then exposed to 40 ng/ml LPS for an additional 12 h. The amount of NO in medium was measured as described in Materials and Methods. (d) Crocetin suppresses LPS-induced iNOS expression. RAW264.7 cells were pretreated with crocetin (10–20 *μ*g/ml) for 1 h before starvation in serum-free medium for 2.5 h and then exposed to 40 ng/ml LPS for an additional 12 h of treatment. The expressions of proteins were detected by Western blot analysis using their corresponding antibodies. Data are presented as the mean ± SD of at least triplicate tests. ^∗^*p* < 0.05 and ^∗∗^*p* < 0.01 vs. LPS-treated cells.

Crocin and crocetin are the main natural pigments that coexisted in Gardenia Yellow with functional potency. It was reported that crocin exerted powerful anti-inflammatory properties by the inhibition of iNOS expression [46]. Therefore, it is necessary to clarify the anti-inflammatory activity of crocetin (the structure is shown in Figure 1(a)). Firstly, the effect of crocetin on the viability of RAW264.7 cells was conducted with the MTT assay. As shown in Figure 1(b), it was found that there is no significant difference in the cell viability when treated with crocetin at the concentrations of 0, 10, 20, and 40 *μ*g/ml, while the number of living cells was significantly reduced at 80 *μ*g/ml (*p* < 0.05). Thus, the concentrations of 10 and 20 *μ*g/ml were selected to proceed in the following study. In Figure 1(c), 40 ng/ml of LPS successfully stimulated the expressions of NO in RAW264.7 cells; pretreatment with 10 or 20 *μ*g/ml of crocetin inhibited the LPS-induced NO release. Moreover, the expression of iNOS, the rate-limiting enzymes of NO, was also detected. As shown in Figure 1(d), dose-dependent inhibition of iNOS expression was found in LPS-stimulated cells after crocetin treatment (*p* < 0.01). These results indicated that crocetin possessed inhibitory activity on NO production and iNOS expression.

### 3.2. Crocetin Inhibits the Activation of the Nuclear Factor kappaB (NF-*κ*B)

**Figure 2:**
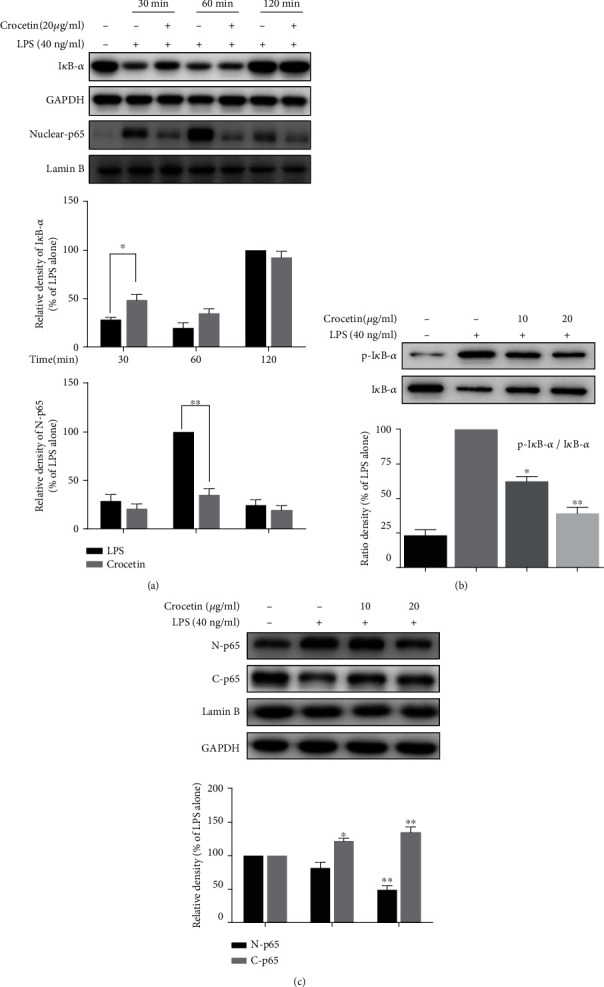
The effect of crocetin on the key nuclear transcription factors of *iNOS* gene. (a) The effect of crocetin on I*κ*B-*α*/p65 expression at an indicated time. RAW264.7 cells were pretreated with crocetin (20 *μ*g/ml) for 1 h before starvation in serum-free medium for 2.5 h and then exposed to 40 ng/ml LPS for an indicated time. The level of protein was detected by Western blot analysis using their corresponding antibodies. (b) Crocetin dose-dependently inhibits LPS-induced degradation of I*κ*B-*α*. RAW264.7 cells were pretreated with crocetin (10–20 *μ*g/ml) for 1 h before starvation in serum-free medium for 2.5 h and then exposed to 40 ng/ml LPS for an additional 30 min. The total and phosphorylation of I*κ*B-*α* were detected by Western blot analysis using their corresponding antibodies. (c) Crocetin inhibits LPS-induced nuclear translocation of p65. Cell culture and crocetin (10–20 *μ*g/ml) treatment were done as described in (b). Extraction of nuclear and cytosolic p65 was performed as described in Materials and Methods. Lamin B and GAPDH were used as a control for nuclear and cytosolic protein, respectively. Data are presented as the mean ± SD of at least triplicate tests. ^∗^*p* < 0.05 and ^∗∗^*p* < 0.01 vs. LPS-treated cells.

To date, transcription factor nuclear factor kappaB (NF-*κ*B) is frequently reported to directly bind to the conserved sequence in the promoter region of *iNOS* gene and can regulate its transcription efficiently [28, 29, 47–49]. Thus, NF-*κ*B was selected in the subsequent study to find which transcriptional factor is the target for crocetin. Firstly, the time course of treatment with 20 *μ*g/ml of crocetin was performed to investigate its effect on the expression of I*κ*B-*α* and p65. As shown in Figure 2(a), 40 ng/ml of LPS was found to significantly reduce the expression of I*κ*B-*α* at 30 min and 60 min (*p* < 0.05) and restore to the basal expression at 120 min; cotreatment with 20 *μ*g/ml of crocetin maintains the high expression of cytoplasm I*κ*B-*α* at 30 min, but not at 60 min and 120 min. Moreover, the expressions of nuclear p65 were inhibited significantly at 60 min by crocetin treatment, compared with LPS stimulation only, while this action disappeared at 120 min. The time course result of LPS treatment only and cotreatment of crocetin indicated that the time points of LPS stimulation in the cell model and the action of the transcription factor are consistent with the previous studies.

To verify the inhibitory effect of crocetin on the NF-*κ*B pathway, the dose course of crocetin treatment was conducted to confirm its effect on the key factor of this pathway, the expressions of cytoplasm I*κ*B-*α* and p-I*κ*B-*α*. The results obtained in Figure 2(b) showed that LPS increased the phosphorylation of I*κ*B-*α* and decreased the protein level of I*κ*B-*α* (*p* < 0.01), and cotreatment with crocetin dose-dependently blocked this process. To further confirm the nuclear translocation of the transcription factor p65, the nuclear translocations of them were both detected. According to the action time point for these two transcription factors in Figure 2(a), the result showed that pretreatment with crocetin can dose-dependently inhibit the LPS-induced nuclear translocation of p65 (Figure 2(c)). All of these results revealed that crocetin inhibited the expression of iNOS by blocking LPS-induced I*κ*B-*α* phosphorylation and p65 translocation.

### 3.3. Crocetin Blocks LPS-Induced iNOS Expression by Increasing MEK1/ERK1 Phosphorylation but Inhibiting JNK Phosphorylation

**Figure 3:**
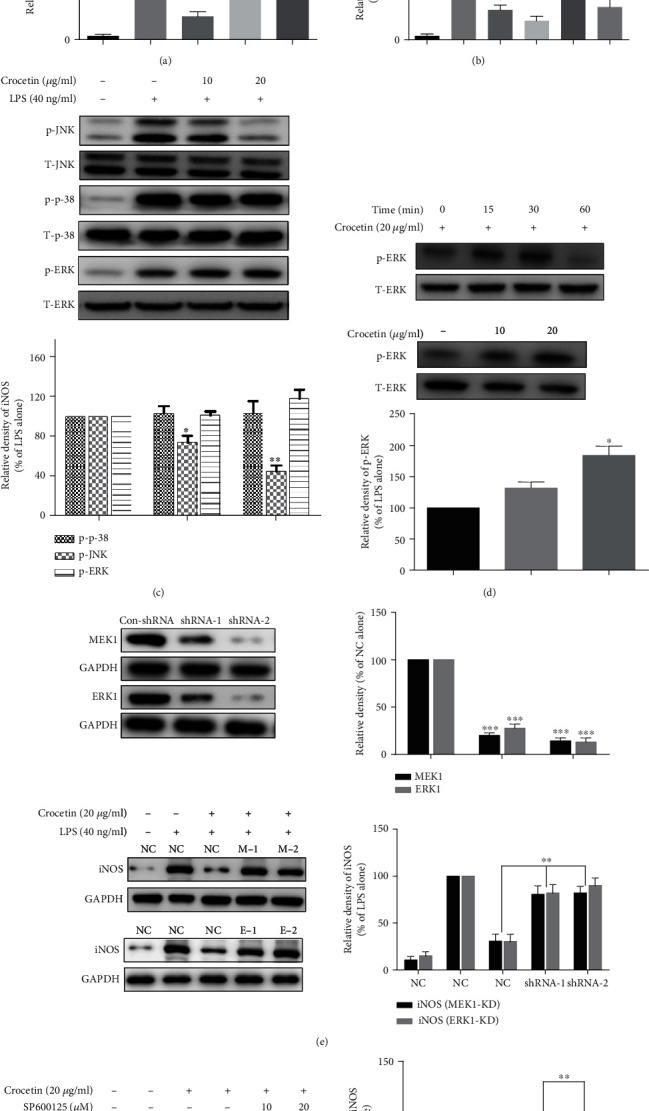
The effect of crocetin on the phosphorylation of protein kinases. (a) JNK inhibitor suppressed LPS-induced iNOS expression. RAW264.7 cells were pretreated for 1 h with AZD6244, SB202190, or SP600125 alone and then exposed to 40 ng/ml LPS for 12 h. The expression of iNOS was detected by Western blot analysis. (b) MEK inhibitor reversed the effect on crocetin-mediated iNOS inhibition. RAW264.7 cells were treated with MAPK inhibitors for 1 h, subsequently added crocetin for another 1 h, and then exposed to 40 ng/ml LPS for 12 h. Western blot was applied to detect iNOS expression. (c) Crocetin inhibited LPS-induced phosphorylation of JNK but has no significant effect on MEK and p38. RAW264.7 cells were pretreated with crocetin (10-20 *μ*g/ml) for 1 h and then exposed to 40 ng/ml LPS for 0.5 h. The total and phosphorylated protein kinases were detected with their antibodies, respectively. (d) MEK1-KD and ERK1-KD relieved the crocetin-induced iNOS inhibition. Selecting RAW264.7 cells with scramble shRNA as blank controls (NC), MEK1-KD cell lines or ERK1-KD cell lines were treated with crocetin for 1 h and then exposed to 40 ng/ml LPS for 12 h. The iNOS expression was detected by Western blot analysis. (e) Crocetin increased the phosphorylation of MEK. RAW264.7 cells were pretreated with crocetin (20 *μ*g/ml) at the indicated time or pretreated with crocetin (10-20 *μ*g/ml) for 30 min, and then, total and phosphorylated protein levels of MEK were detected with their antibodies, respectively. (f) JNK is critical for LPS-induced iNOS expression. ERK1-KD (shRNA-2) cells were pretreated with SP600125 (10-20 *μ*M) for 1 h, and the following steps were done as described in (a). The expressions of iNOS were detected by Western blot analysis. Each value represents the mean ± SD of triplicate tests. ^∗^*p* < 0.05 and ^∗∗^*p* < 0.01 vs. LPS-treated cells.

MAPKs play essential roles in LPS or phytochemical stimulated iNOS expression in RAW264.7 cells; thus, it is necessary to detect the protein kinase cascades and their activities in the crocetin-induced NO inhibitory effect to better elucidate the underlying mechanism. Firstly, several typical protein kinase inhibitors were selected to be applied in the following experiment, including AZD6244, SB202190, and SP600125, which are the inhibitors for MEK1/2, p38 kinase, and JNK, respectively. As shown in Figure 3(a), pretreatment of JNK inhibitor can markedly reduce LPS-induced iNOS expression (*p* < 0.001), MEK inhibitor only slightly reduce that, and p38 has no effect, which implied that only JNK is essential in LPS-induced iNOS expression. To further investigate the coeffect of crocetin on this process, 20 *μ*g/ml crocetin was coadded in the cells with protein kinase inhibitor treatment. Surprisingly, as shown in Figure 3(b), pretreatment with MEK inhibitor eliminated the inhibitory effect of crocetin on iNOS expression (*p* < 0.01). These results indicated that crocetin may inhibit the expression of iNOS by regulating the phosphorylation of MEK and JNK.

Moreover, to investigate the actual effect of crocetin on the protein kinase pathways of JNK, MEK, and p38, a series of immune blots were detected. As shown in Figure 3(c), crocetin only showed a dose-dependent inhibitory effect on LPS-induced phosphorylation of JNK (*p* < 0.01), but there is no significant effect on phosphorylation of MEK and p38. This result aroused our curiosity, and we speculate that the reason for this phenomenon is that the MEK inhibitor has diverse targets or mutual antagonism competing with crocetin. Subsequently, shRNA technology was applied to knock down (KD) the gene expression of *MEK1* and *ERK1* to eliminate these potential possibilities. Interestingly, the same result was obtained as MEK inhibitor treatment (Figure 3(b)); both MEK1 knockdown (MEK1-KD) and ERK knockdown (ERK1-KD) restored LPS-induced iNOS expression which is inhibited by crocetin treatment, *p* < 0.01 (Figure 3(e)). These results indicated that the above possibilities are not the reason for the not significant effect of crocetin on the MEK pathway; maybe the treatment time of its inhibitor for a long time is the cause. Therefore, pretreatment with crocetin for 15-60 min was selected for immunoblot, instead of the previous 90 min (Figure 3(c)). As shown in Figure 3(d), it was found that the phosphorylation of ERK was increased significantly within 30 minutes after crocetin treatment, and crocetin also increased the expressions of p-ERK in a dose-dependent manner (*p* < 0.05).

Furthermore, to investigate the relationship between MEK1/ERK1 and JNK, the JNK inhibitor was used in ERK1-KD cells. The result showed that the JNK inhibitor can still significantly inhibit the expression of iNOS in the ERK1-KD cell line in a dose-dependent manner, *p* < 0.001 (Figure 3(f)), which indicated that JNK played a crucial role in LPS-stimulated iNOS expression, and crocetin exerted its anti-inflammatory effect via induction of MEK1/ERK1 phosphorylation and subsequent inhibition of JNK phosphorylation.

### 3.4. Crocetin Exerts Its Anti-inflammatory Effect via Activation of Nrf2/HO-1 Signaling Pathway

**Figure 4:**
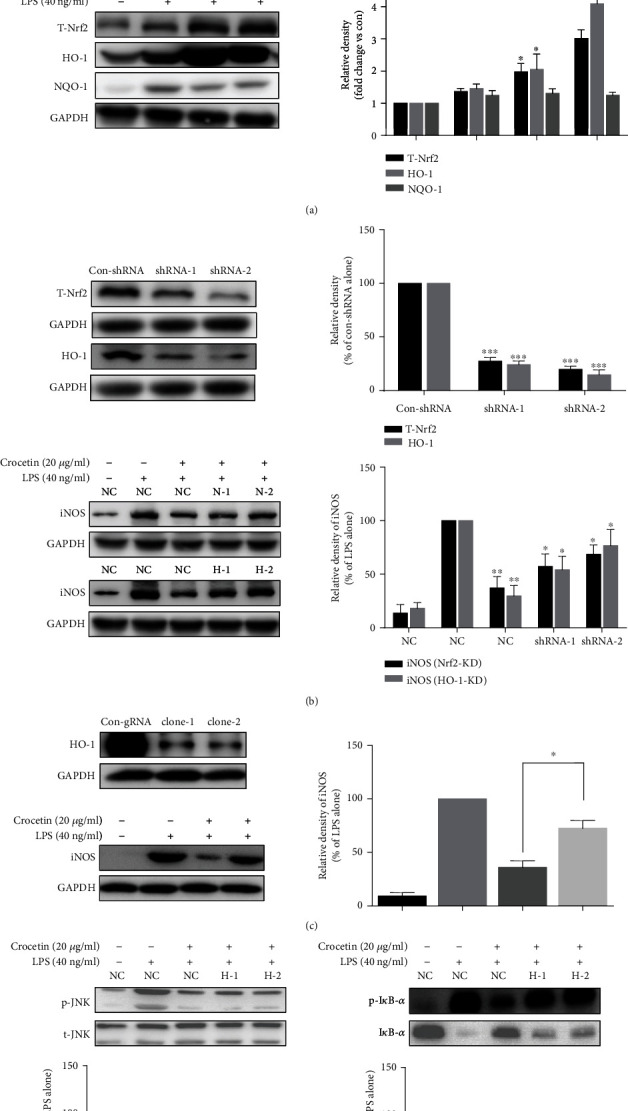
The involvement of the Nrf2/HO-1 pathway in the crocetin-induced inhibitory activity on iNOS expression. (a) Crocetin increased the expressions of Nrf2 and HO-1 and the nuclear translocation of Nrf2. For Nrf2 and HO-1 expressions, RAW264.7 cells were pretreated with the indicated dose of crocetin for 1 h before starvation in serum-free medium for 2.5 h and then exposed to 40 ng/ml LPS for an indicated time. For Nrf2 nuclear translocation, cell culture and crocetin (20 *μ*g/ml) treatment were done as described in Figure 2(c). The expressions of proteins were detected by Western blot analysis using their corresponding antibodies. (b) Nrf2-knockdown (Nrf2-KD) and HO-1-knockdown (HO-1-KD) blocked crocetin-dependent iNOS inhibition. Detection of the knockout efficiency of two monoclonal cells (top). NC and HO-1-KO cell treatment was done as described in (b). (c) HO-1-knockout (HO-1-KO) blocked crocetin-dependent iNOS inhibition. The total and phosphorylated protein kinases were detected with their antibodies, respectively. (d) HO-1-KO blocked crocetin-dependent p-I*κ*B-*α* inhibition. NC and HO-1-KO cell treatment was done as described in Figure 2(b). Data are presented as the mean ± SD of triplicate tests. ^∗^*p* < 0.05 and ^∗∗^*p* < 0.01 vs. LPS-treated cells.

It has been reported that the activation of the Nrf2/HO-1 pathway is involved in the attenuation of NF-*κ*B translocation and iNOS expression [50, 51]. To investigate the effect of crocetin on the crosstalk between Nrf2/HO-1 and NF-*κ*B/iNOS pathway and whether this crosstalk is essential for its anti-inflammatory property, gene knockdown and knockout technologies had been performed. As shown in Figure 4(a), the time course result of 4-16 h showed that the expressions of total Nrf2 and its downstream antioxidant protein HO-1 were increased significantly by crocetin treatment (*p* < 0.01), but not that by NQO1, compared with LPS treatment only; the time course result of 30-120 min showed that the expressions of both cytoplasm and nuclear Nrf2 were induced significantly by crocetin treatment (*p* < 0.01). Besides, crocetin was found to activate the Nrf2/HO-1 pathway significantly in a dose-dependent manner.

To verify the essential role of the Nrf2/HO-1 pathway in crocetin-inhibited iNOS expression, both *Nrf2* and *HO-1* genes were knocked down. As shown in Figure 4(b), two repeated KD genes for *Nrf2* and *HO-1* had been constructed (*p* < 0.001), and the results showed that knockdown of *HO-1* and *Nrf2* genes could largely diminish the inhibitory effect of crocetin on LPS-induced iNOS overexpression, *p* < 0.05 (Figure 4(b)). Moreover, the CRISPR/Cas9 system was used to construct the *HO-1* gene knockout (KO) cell model to further confirm this action. As shown in Figure 4(c), the results of *HO-1*-KO cells displayed are similar to that of *HO-1*-KD cells (Figure 4(b)). Further, the role of HO-1 in the inflammatory signaling pathway was determined. As shown in Figure 4(d), crocetin significantly inhibited the expression of p-I*κ*B-*α* (*p* < 0.01), but this effect can be reversed in HO-1-KO cells. In addition, crocetin significantly inhibited the phosphorylation of JNK, and this effect can also be detected in HO-1-KO cells (*p* < 0.01). These results strengthen the direct crosstalk between the Nrf2/HO-1 and NF-*κ*B/iNOS pathways and highlight the essential role of HO-1 in this crosstalk.

The above data indicated that crocetin may exert its anti-inflammatory effect via activation of the Nrf2/HO-1 signaling pathway, and HO-1 is a vital link to regulate the redox balance in RAW264.7 cells treated with crocetin.

### 3.5. The Direct Binding Proof and Docking Model of Crocetin to MEK1, JNK1/2, Keap1-Nrf2, and TLR4-MD2

**Figure 5:**
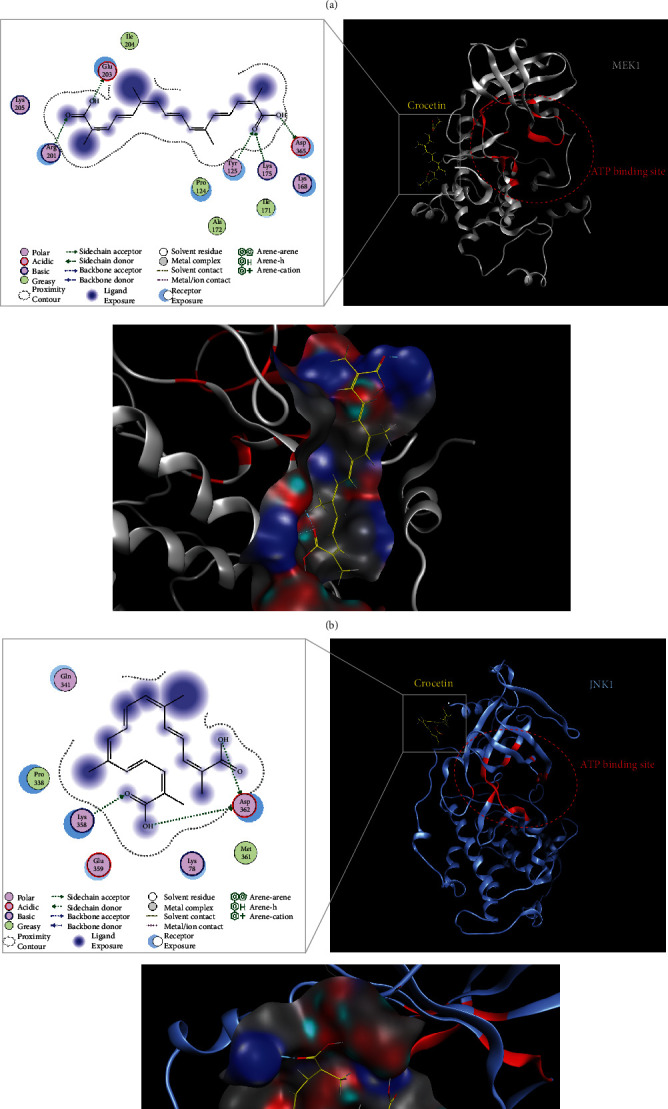
The direct binding proof and computational docking model for the action of crocetin. (a) Binding abilities of crocetin to MEK1, JNK1/2, and Keap1-Nrf2. Whole cell lysate (input control, lane 1), lysate precipitation with Sepharose 4B beads (negative control, lane 2), and Sepharose 4B-crocetin-coupled beads (lane 3) were applied to SDS-PAGE and then detected with MEK1, JNK1/2, Nrf2, or Keap1 antibody, respectively. The binding efficiency to crocetin was presented as the ratio of input control. (b, c) The docking models of crocetin to MEK1 and JNK1/2. Electrostatic potential surface is indicated in a close-up figure of the upper side, and hydrogen bonds are indicated by blue and green lines in the lower figure. White, blue ribbon: protein kinase; red ribbon: ATP-binding site; yellow: crocetin.

Our data suggest that MEK1, JNK1/2, Keap1, and Nrf2 are potential targets for crocetin to inhibit inflammatory signaling. Thus, we investigated whether the crocetin directly binds to them, using the bead-bound pull-down assay, which has been validated as an effective screening tool in our previous study [42]. In brief, crocetin is coupled with CNBr-Sepharose 4B beads and then incubated with protein lysate extracted from RAW264.7 cells. The bound protein was extracted by centrifuge and detected by Western blotting with respective antibodies after washing out. As shown in Figure 5(a), MEK1 and JNK1/2 were detected in the complex of Sepharose 4B beads-crocetin-proteins, with 22.7% and 95% binding rates compared to the cell lysate control, while only JNK1/2 was slightly detected in the Sepharose 4B beads alone. Meanwhile, Keap1, the major Nrf2 endogenous inhibitor, has shown a powerful combination with Crocetin-Sepharose 4B beads with a 194.6% binding rate, and only 12.5% binding rate was detected with Nrf2 antibody, while both Keap1 and Nrf2 were not detected in the Sepharose 4B beads alone. These data showed that crocetin could bind directly with protein kinases of MEK1 and JNK1/2. Moreover, Keap1 had stronger binding activity than Nrf2, which is suggested that crocetin may directly combine with Keap1 for modification and then release Nrf2 from the Keap1-Nrf2 complex.

As shown in Supplementary Fig. 3A, the computational docking analysis data showed that there are 3 hydrogen bonds for the binding of crocetin to the TLR4-MD2 complex, which is not the same binding region of LPS. This result suggested that crocetin may not bind competitively with LPS to block the LPS-induced inflammation. To know how crocetin binds to MEK1, JNK1/2, and Keap1-Nrf2 complex, the computer modeling of crocetin bound to these proteins was performed, using the software as described in Materials and Methods. The results provided interesting information that five hydrogen bonds were formed between crocetin and Arg201, Glu201, Tyr125, Lys175, and Asp635 residues of MEK1, which are configured nearby ATP-binding pocket (Figure 5(b)). As shown in Figure 5(c), three hydrogen bonds were formed between crocetin and Lys358 and Asp362 residues of JNK1/2, which configured a part of the ATP-binding pocket. Further analysis data suggested that crocetin can directly bind to MEK1 on the ATP-binding site 145 and 146 and bind to JNK1/2 by hydrogen bond 358. For the Keap1-Nrf2 complex, three hydrogen bonds were formed between crocetin and His552, Asp579, and Asp573 residues of Keap1, which did not configure a part of the Keap1 catalytic domain (Supplementary Fig. 3B). These docking results further support our pull-down binding data between crocetin and MEK1 and JNK1/2.

## 4. Discussion

**Figure 6:**
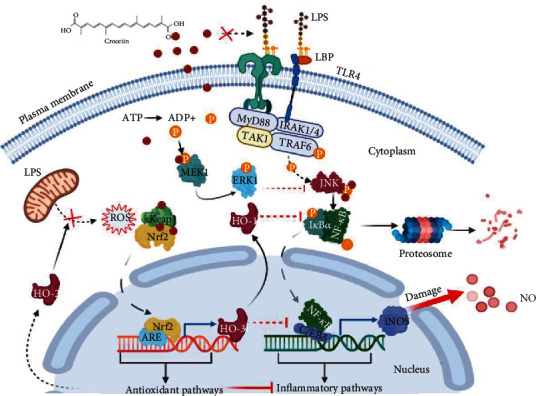
Schematic diagram of the molecular mechanism underlying the anti-inflammatory effect of crocetin on LPS-induced RAW264.7 cells. LPS can stimulate the activation of the TLR4/NF-*κ*B/iNOS inflammatory pathway in RAW264.7 cells. Crocetin treatment effectively inhibits LPS-induced iNOS expression by inhibition of the MEK1/ERK1/JNK/NF-*κ*B/iNOS pathway and activation of the Nrf2/HO-1 pathway. HO-1 is the link point for the crosstalk between these two pathways, and KD or KO of *HO-1* gene can eliminate the potent anti-inflammatory effect of crocetin. In addition, crocetin treatment significantly inhibits the LPS-induced ROS production, while this protective mechanism involved is not clarified, which is involved in protected effects.

Crocetin is previously reported as an effective anti-inflammatory agent in other studies [25, 52]. However, the molecular mechanism underlying this action has not been fully elucidated, especially the effect of crocetin on the extracellular receptor and the crosstalk between inflammatory pathways and other pathways. In the present study, the molecular mechanism of the anti-inflammatory property of crocetin in the LPS-stimulated RAW264.7 cell model had been investigated in depth. The results showed that crocetin exerts its anti-inflammatory property mainly via the MEK1/ERK1/JNK/NF-*κ*B/iNOS pathway (Figure 1). Further molecular data by application of shRNA-KD and CRISPR-Cas9 KO technologies confirmed the anti-inflammatory effect of crocetin, and what is more interesting, KD and KO of *HO-1* gene both inhibited this action (Figure 4). Coupled with the molecular data of Nrf2 activation by crocetin, the present study discovers the crosstalk between the Nrf2/HO-1 pathway and the MEK1/ERK1/JNK/NF-*κ*B/iNOS pathway, and HO-1 plays an essential role in the regulatory effect of crocetin on keeping the redox balance (Figures 3 and 4). The molecular mechanism in detail is summarized in Figure 6.

NO, as a major inflammatory mediator, has played a significant role in the expansion of oncogenesis and inflammatory reaction, and it is suggested that the inhibition of NO overproduction or downregulation of iNOS is an imperative target for the prevention and treatment of inflammation and its related complications. Up to now, several studies, both in vitro and in vivo, have shown that crocetin has strong anti-inflammatory activity via inhibition of NO or iNOS in cancer cells [46, 53]. However, the effect of crocetin on the above two inflammatory mediators in the RAW cell model has not been fully investigated [25]. In the present study, it was found that the anti-inflammatory property of crocetin depends on its inhibition of NO and iNOS.

Mitogen-activated protein kinase activation induces the expression of multiple inflammatory genes by regulating nuclear transcription factor NF-*κ*B [54, 55]. In the present study, specific inhibitors of these protein kinases were used, and it was found that only JNK and MEK1/ERK1 are associated with the anti-inflammatory activity of crocetin (Figure 3). Further data by using MEK1/ERK1-KD cells and JNK inhibitor implied that phosphorylation of JNK is essential for the action of crocetin, and JNK is downstream of MEK1/ERK1, which is consistent with other previous studies [54, 56, 57]. In brief, it was reported that dual-specificity protein phosphatases (DUSP) can dephosphorylate both the threonine and tyrosine residues in the activation loop of MAPKs, thereby inactivating them [56]. Activation of MEK1/ERK1 can increase the transcription of DUSP2 which can limit JNK phosphorylation [57]. More powerful evidences have been shown by using Sepharose 4B beads and pull-down assay to find out the direct binding proof of crocetin to MEK1 and JNK1/2. Our result (Figure 5(a)) suggested that crocetin may directly bind to MEK1 and JNK1/2. Besides, computational docking of crocetin to MEK1 and JNK1/2 (Figures 5(b) and 5(c)) was also analyzed, and the results provided some interesting binding information between the hydroxyl groups of crocetin and amino acid residues of MEK1 and JNK1/2.

As is well known, the Nrf2/HO-1 antioxidant signaling pathway plays a crucial role in the anti-inflammatory process. LPS can stimulate a higher level of ROS generation, cytokines, and chemokines in Nrf2-KO cells [58, 59]. Several studies have reported the crosstalk between Nrf2/HO-1 with NF-*κ*B/iNOS. Nrf2-knockout mice showed increased mRNA and protein levels of iNOS, and the activation of Nrf2 leads to the reduction of iNOS [58, 60]. Nrf2 or HO-1 gene-deficient mice with pneumococcal meningitis showed significantly higher levels of HMGB1 and iNOS [61]. Here, the data showed that crocetin can activate the Nrf2/HO-1 pathway (Figure 4(a)) which may be attributed to the direct binding between crocetin and Keap1-Nrf2 complex (Supplementary Fig. 3B). Nrf2-KD, HO-1-KD, and HO-1-KO inhibition can attenuate crocetin-mediated iNOS inhibitory activity (Figures 4(b) and 4(c)). More detail is shown in Figure 4(d) that crocetin had increased the expression of HO-1 which is contributed to the stability of I*κ*B-*α* (Figure 4(d)). This result was also well replicated in another study [62]. These data implied that this action of crocetin is partially attributed to the activation of the Nrf2/HO-1 pathway, especially the induction of HO-1. Moreover, a series of studies speculate that the regulation of iNOS by the Nrf2/HO-1 antioxidant signaling pathway stems from its ability to inhibit the production of ROS which can effectively activate MAPKs [63]. However, as shown in Supplementary Fig. 1, crocetin was found to dose-dependently inhibit LPS-induced ROS production, but treatment with different concentrations of NAC, a common ROS scavenger [64], has no effect on LPS-induced iNOS expression. These results suggested that there was no significant correlation between the crocetin-mediated iNOS inhibitory activity and the reduction of crocetin-mediated ROS production. Thus, further research is needed to elucidate the molecular mechanism in depth underlying the relationship between ROS, Nrf2/HO-1, and NF-*κ*B/iNOS.

A previous review suggests that the immunomodulatory activity of crocetin may be caused by direct targeting Toll-like receptors (TLRs) and the subsequent regulation of various transcription factors such as NF-*κ*B and AP-1 [65]. Thus, to confirm whether crocetin competes with LPS for binding to TLR4 to exert its anti-inflammatory property, computational docking analysis based on the structure of the TLR4-MD2 complex (PDB ID: 5IJB) and crocetin was performed. The data (Supplementary Figure 3A) showed that crocetin is docked in the different domains of the TLR4-MD2 complex with LPS, suggesting that crocetin might have no competitive binding to the TLR4-MD2 complex with LPS. Moreover, another piece of evidence is that knockdown of TLR-4 has no effect on crocetin-caused induction of HO-1 (Supplementary Fig. 2). These data further confirmed that crocetin exerts its anti-inflammatory effect by not targeting TLR-4, and which potent extracellular receptors crocetin targets require more detailed exploration.

## 5. Conclusion

In the present study, it was found that crocetin exerted its anti-inflammatory property by inhibiting the MEK1/JNK/NF-*κ*B/iNOS pathway and activating the Nrf2/HO-1 pathway; the direct crosstalk between the MEK1/JNK/NF-*κ*B/iNOS pathway and the Nrf2/HO-1 pathway is existing in crocetin-treated cells, which is essential for the anti-inflammatory effect of crocetin; HO-1 is the key link point of the above crosstalk. Therefore, crocetin can act as a redox balance modulator to orchestrate precisely its anti-inflammatory and chemopreventive effect via its regulatory action on the crosstalk between the NF-*κ*B/iNOS pathway and the Nrf2/HO-1 pathway.

## Data Availability

The authors confirm that all data underlying the findings are available. All relevant data are within the paper and its supporting information files.

## Supplementary Materials

Supplementary 1 Supplementary Figure 1: the role of ROS in LPS-induced iNOS expression. (A) Crocetin inhibits LPS-induced ROS production. Cell culture and treatment were done as described in Materials and Methods. (B) NAC (ROS scavengers) cannot inhibit LPS-induced iNOS expression. RAW264.7 cells were treated with NAC (5–15 *μ*M) for 1 h before starvation in serum-free medium for another 2.5 h and then exposed to 40 ng/ml LPS for an additional 12 h. The expressions of iNOS were detected by Western blot analysis. Data are presented as the mean ± SD of at least triplicate tests. ^∗^*p* < 0.05 and ^∗∗^*p* < 0.01 vs. LPS-treated cells.

Supplementary 2 Supplementary Figure 2: the involvement of TLR-4 in iNOS and HO-1 expressions. (A) RT-qPCR test of gene knockdown efficiency. (B) TLR-4 is involved in LPS-induced iNOS inhibition but not involved in crocetin-induced HO-1 overexpression. NC and TLR-4-KD cells were pretreated with crocetin (20 *μ*g/ml) for 1 h and then exposed to 40 ng/ml LPS for an additional 12 h. The HO-1 and iNOS expression was detected by Western blot analysis using their corresponding antibodies. Data are presented as the mean ± SD of triplicate tests. ^∗^*p* < 0.05 and ^∗∗^*p* < 0.01 vs. LPS-treated cells.

Supplementary 3 Supplementary Figure 3: the docking models of crocetin to TLR4-MD2 and Keap1-Nrf2 complex. (A) There are 3 hydrogen bonds for the binding of crocetin to the TLR4-MD2 complex, which is not the same binding region of LPS. (B) For the Keap1-Nrf2 complex, three hydrogen bonds were formed between crocetin and His552, Asp579, and Asp573 residues of Keap1, which did not configure a part of the Keap1 catalytic domain.

## References

[1] Higashino S., Sasaki Y., Giddings J. C. (2014). Crocetin, a carotenoid from gardenia jasminoides Ellis, protects against hypertension and cerebral thrombogenesis in stroke-prone spontaneously hypertensive rats. *Phytotherapy Research*.

[2] Hashemi M., Hosseinzadeh H. (2019). A comprehensive review on biological activities and toxicology of crocetin. *Food and Chemical Toxicology*.

[3] Rameshrad M., Razavi B. M., Hosseinzadeh H. (2018). Saffron and its derivatives, crocin, crocetin and safranal: a patent review. *Expert Opinion on Therapeutic Patents*.

[4] Dong L. C., Fan Y. X., Yu Q. (2015). Synergistic effects of rhubarb-gardenia herb pair in cholestatic rats at pharmacodynamic and pharmacokinetic levels. *Journal of Ethnopharmacology*.

[5] Chen H., Huang X., Min J. (2016). Geniposidic acid protected against ANIT-induced hepatotoxity and acute intrahepatic cholestasis, due to Fxr-mediated regulation of Bsep and Mrp2. *Journal of Ethnopharmacology*.

[6] Zhang H., Lai Q., Li Y., Liu Y., Yang M. (2017). Learning and memory improvement and neuroprotection of *Gardenia jasminoides* (*Fructus gardenia*) extract on ischemic brain injury rats. *Journal of Ethnopharmacology*.

[7] Song X., Zhang W., Wang T. (2014). Geniposide plays an anti-inflammatory role via regulating TLR4 and downstream signaling pathways in lipopolysaccharide-induced mastitis in mice. *Inflammation*.

[8] Xiaofeng Y., Qinren C., Jingping H. (2012). Geniposide, an iridoid glucoside derived from *Gardenia jasminoides*, protects against lipopolysaccharide-induced acute lung injury in mice. *Planta Medica*.

[9] Zhang C., Li Y. T., Xiao Y. Q., Yu D. R., Ma Y. L., Gu X. Z. (2013). Content comparison of main chemical compositions in Gardenia jasminoids roasted with ginger juice. *China Journal of Chinese Materia Medica*.

[10] Zhao C., Zhang H., Li H. (2017). Geniposide ameliorates cognitive deficits by attenuating the cholinergic defect and amyloidosis in middle-aged Alzheimer model mice. *Neuropharmacology*.

[11] Soltani F., Ramezani M., Amel Farzad S., Mokhtarzadeh A., Hashemi M. (2017). Comparison study of the effect of alkyl-modified and unmodified PAMAM and PPI dendrimers on solubility and antitumor activity of crocetin. *Artificial Cells, Nanomedicine, and Biotechnology*.

[12] Zhang A., Li J. (2017). Crocetin shifts autophagic cell survival to death of breast cancer cells in chemotherapy. *Tumor Biology*.

[13] Li S., Qu Y., Shen X. Y. (2019). Multiple signal pathways involved in crocetin-induced apoptosis in KYSE-150 cells.. *Pharmacology*.

[14] Nam K. N., Park Y.-M., Jung H.-J. (2010). Anti-inflammatory effects of crocin and crocetin in rat brain microglial cells. *European Journal of Pharmacology*.

[15] Lautenschläger M., Sendker J., Hüwel S. (2015). Intestinal formation of *trans*-crocetin from saffron extract (*Crocus sativus*L.) and *in vitro* permeation through intestinal and blood brain barrier. *Phytomedicine*.

[16] Wang X., Jiao X., Liu Z., Li Y. (2017). Crocetin potentiates neurite growth in hippocampal neurons and facilitates functional recovery in rats with spinal cord injury. *Neuroscience Bulletin*.

[17] Li S. (2017). A corpus-based study of vague language in legislative texts: strategic use of vague terms. *English for Specific Purposes*.

[18] Song L., Kang C., Sun Y., Huang W., Liu W., Qian Z. (2016). Crocetin inhibits lipopolysaccharide-induced inflammatory response in human umbilical vein endothelial cells. *Cellular Physiology and Biochemistry*.

[19] Chen P., Chen Y., Wang Y. (2016). Comparative evaluation of hepatoprotective activities of geniposide, crocins and crocetin by CCl4-induced liver injury in mice. *Biomolecules & Therapeutics*.

[20] Yang R., Vernon K., Thomas A., Morrison D., Qureshi N., van Way C. W. (2011). Crocetin reduces activation of hepatic apoptotic pathways and improves survival in experimental hemorrhagic shock. *Journal of Parenteral and Enteral Nutrition*.

[21] Xiang M., Yang R., Zhang Y. (2017). Effect of crocetin on vascular smooth muscle cells migration induced by advanced glycosylation end products. *Microvascular Research*.

[22] Mahdavifard S., Bathaie S. Z., Nakhjavani M., Taghikhani M. (2016). The synergistic effect of antiglycating agents (MB-92) on inhibition of protein glycation, misfolding and diabetic complications in diabetic- atherosclerotic rat. *European Journal of Medicinal Chemistry*.

[23] Yang L., Qian Z., Ji H. (2010). Inhibitory effect on protein kinase C*θ* by Crocetin attenuates palmitate- induced insulin insensitivity in 3T3-L1 adipocytes. *European Journal of Pharmacology*.

[24] Inoue E., Shimizu Y., Shoji M., Tsuchida H., Sano Y., Ito C. (2005). Pharmacological properties of N-095, a drug containing red ginseng, polygala root, saffron, antelope horn and aloe wood. *American Journal of Chinese Medicine*.

[25] Chen B., Hou Z. H., Dong Z., Li C. D. (2015). Crocetin Downregulates the Proinflammatory Cytokines in Methylcholanthrene- Induced Rodent Tumor Model and Inhibits COX-2 Expression in Cervical Cancer Cells. *BioMed Research International*.

[26] Ignarro L. J. (2002). Nitric oxide as a unique signaling molecule in the vascular system: a historical overview. *Journal of Physiology and Pharmacology*.

[27] Prast H., Philippu A. (2001). Nitric oxide as modulator of neuronal function. *Progress in Neurobiology*.

[28] Bhat N. R., Feinstein D. L., Shen Q., Bhat A. N. (2002). p38 MAPK-mediated Transcriptional activation of inducible nitric-oxide synthase in glial cells:. *Journal of Biological Chemistry*.

[29] Saha R. N., Pahan K. (2006). Regulation of inducible nitric oxide synthase gene in glial cells. *Antioxidants & Redox Signaling*.

[30] Zhang X., Ding M., Zhu P. (2019). New insights into the Nrf-2/HO-1 signaling axis and its application in pediatric respiratory diseases. *Oxidative Medicine and Cellular Longevity*.

[31] Willis D., Moore A. R., Frederick R., Willoughby D. A. (1996). Heme oxygenase: a novel target for the modulation of inflammatory response. *Nature Medicine*.

[32] Minamino T., Christou H., Hsieh C.-M. (2001). Targeted expression of heme oxygenase-1 prevents the pulmonary inflammatory and vascular responses to hypoxia. *Proceedings of the National Academy of Sciences of the United States of America*.

[33] Lee T. S., Chau L. Y. (2002). Heme oxygenase-1 mediates the anti-inflammatory effect of interleukin-10 in mice. *Nature Medicine*.

[34] Poss K. D., Tonegawa S. (1997). Heme oxygenase 1 is required for mammalian iron reutilization. *Proceedings of the National Academy of Sciences of the United States of America*.

[35] Yachie A., Niida Y., Wada T. (1999). Oxidative stress causes enhanced endothelial cell injury in human heme oxygenase-1 deficiency. *Journal of Clinical Investigation*.

[36] Tsoyi K., Nizamutdinova I. T., Jang H. J. (2010). Carbon monoxide from CORM-2 reduces HMGB1 release through regulation of IFN-*β*/JAK2/STAT-1/INOS/NO signaling but not COX-2 in TLR-activated macrophages. *Shock*.

[37] Park E. J., Kim Y. M., Kim H. J., Chang K. C. (2018). Luteolin activates ERK1/2- and Ca2+-dependent HO-1 induction that reduces LPS-induced HMGB1, iNOS/NO, and COX-2 expression in RAW264.7 cells and mitigates acute lung injury of endotoxin mice. *Inflammation Research*.

[38] Kirikae T., Tamura H., Hashizume M. (1997). Endotoxin contamination in fetal bovine serum and its influence on tumor necrosis factor production by macrophage-like cells J774.1 cultured in the presence of the serum. *International Journal of Immunopharmacology*.

[39] Yang Z., Khemlani L. S., Dean D. F., Carter C. D., Slauson D. O., Bochsler P. N. (1994). Serum components enhance bacterial lipopolysaccharide-induced tissue factor expression and tumor necrosis factor-alpha secretion by bovine alveolar macrophages in vitro. *Journal of Leukocyte Biology*.

[40] Jian Z. J., Yang Z., Mason G. L., Slauson D. O., Bochsler P. N. (1995). Regulation of superoxide anion generation in bovine alveolar macrophages by bacterial lipopolysaccharide, serum proteins, and modulators of signal transduction. *Inflammation*.

[41] Uto T., Fujii M., Hou D. X. (2005). Inhibition of lipopolysaccharide-induced cyclooxygenase-2 transcription by 6-(methylsulfinyl) hexyl isothiocyanate, a chemopreventive compound from *Wasabia japonica* (Miq.) Matsumura, in mouse macrophages. *Biochemical Pharmacology*.

[42] Hisanaga A., Mukai R., Sakao K., Terao J., Hou D.-X. (2016). Anti-inflammatory effects and molecular mechanisms of 8-prenyl quercetin. *Molecular Nutrition and Food Research*.

[43] Kumamoto T., Fujii M., Hou D. X. (2009). Akt is a direct target for myricetin to inhibit cell transformation. *Molecular and Cellular Biochemistry*.

[44] Swiech L., Heidenreich M., Banerjee A. (2015). *In vivo* interrogation of gene function in the mammalian brain using CRISPR-Cas9. *Nature Biotechnology*.

[45] Lebel C. P., Ischiropoulos H., Bondy S. C. (1992). Evaluation of the probe 2′,7′-dichlorofluorescin as an indicator of reactive oxygen species formation and oxidative stress. *Chemical Research in Toxicology*.

[46] Hong Y. J., Yang K. S. (2013). Anti-inflammatory activities of crocetin derivatives from processed Gardenia jasminoides. *Archives of Pharmacal Research*.

[47] Baeuerle P., Baltimore D. (1988). I kappa B: a specific inhibitor of the NF-kappa B transcription factor. *Science*.

[48] Lekstrom-Himes J., Xanthopoulos K. G. (1998). Biological role of the ccaat/enhancer-binding protein family of transcription factors. *Journal of Biological Chemistry*.

[49] Won J. S., Im Y. B., Key L., Singh I., Singh A. K. (2003). The involvement of glucose metabolism in the regulation of inducible nitric oxide synthase gene expression in glial cells: possible role of glucose-6-phosphate dehydrogenase and CCAAT/enhancing binding protein. *Journal of Neuroscience*.

[50] Lee W., Yang S., Lee C. (2019). Aloin reduces inflammatory gene iNOS via inhibition activity and p-STAT-1 and NF-*κ*B. *Food and Chemical Toxicology*.

[51] Luo D., Guo Y., Cheng Y., Zhao J., Wang Y., Rong J. (2017). Natural product celastrol suppressed macrophage M1 polarization against inflammation in diet-induced obese mice via regulating Nrf2/HO-1, MAP kinase and NF-*κ*B pathways. *Aging*.

[52] Yang R., Tan X., Thomas A. M. (2006). Crocetin inhibits mRNA expression for tumor necrosis Factor‐*α*, Interleukin‐1*β*, and inducible nitric oxide synthase in hemorrhagic shock. *Journal of Parenteral & Enteral Nutrition*.

[53] Li Y., Kakkar R., Wang J. (2018). In vivo and in vitro approach to anti-arthritic and anti-inflammatory effect of crocetin by alteration of nuclear factor-E2-related factor 2/hem oxygenase (HO)-1 and NF-*κ*B expression. *Frontiers in Pharmacology*.

[54] Arthur J. S., Ley S. C. (2013). Mitogen-activated protein kinases in innate immunity. *Nature Reviews Immunology*.

[55] Li Y., Zou L., Li T., Lai D., Wu Y., Qin S. (2019). Mogroside V inhibits LPS-induced COX-2 expression/ROS production and overexpression of HO-1 by blocking phosphorylation of AKT1 in RAW264.7 cells. *Acta Biochimica et Biophysica Sinica*.

[56] Caunt C. J., Keyse S. M. (2013). Dual-specificity MAP kinase phosphatases (MKPs): shaping the outcome of MAP kinase signalling. *The FEBS Journal*.

[57] Jeffrey K. L., Brummer T., Rolph M. S. (2006). Positive regulation of immune cell function and inflammatory responses by phosphatase PAC-1. *Nature Immunology*.

[58] Thimmulappa R. K., Scollick C., Traore K. (2006). Nrf2-dependent protection from LPS induced inflammatory response and mortality by CDDO-Imidazolide. *Biochemical and Biophysical Research Communications*.

[59] Luo J.-F., Shen X.-Y., Lio C. K. (2018). Activation of Nrf2/HO-1 pathway by Nardochinoid C inhibits inflammation and oxidative stress in lipopolysaccharide-stimulated macrophages. *Frontiers in Pharmacology*.

[60] Ho F. M., Kang H. C., Lee S. T. (2007). The anti-inflammatory actions of LCY-2-CHO, a carbazole analogue, in vascular smooth muscle cells. *Biochemical Pharmacology*.

[61] Li Z., Ma Q. Q., Yan Y. (2016). Edaravone attenuates hippocampal damage in an infant mouse model of pneumococcal meningitis by reducing HMGB1 and iNOS expression via the Nrf2/HO-1 pathway. *Acta Pharmacologica Sinica*.

[62] Thimmulappa R. K., Lee H., Rangasamy T. (2006). Nrf2 is a critical regulator of the innate immune response and survival during experimental sepsis. *Journal of Clinical Investigation*.

[63] Rahman I., Adcock I. M. (2006). Oxidative stress and redox regulation of lung inflammation in COPD. *The European Respiratory Journal*.

[64] Suzuki M., Bandoski C., Bartlett J. D. (2015). Fluoride induces oxidative damage and SIRT1/autophagy through ROS-mediated JNK signaling. *Free Radical Biology and Medicine*.

[65] Zeinali M., Zirak M. R., Rezaee S. A., Karimi G., Hosseinzadeh H. (2019). Immunoregulatory and anti-inflammatory properties of *Crocus sativus* (Saffron) and its main active constituents: a review. *Iranian Journal of Basic Medical Sciences*.

